# Supplementary material for: The relationship of recreational runners’ motivation and resilience levels to the incidence of injury: A mediation model
Source: PLoS One. 2020 May 11;15(5):e0231628. doi: 10.1371/journal.pone.0231628 (PMC7213716; doi:10.1371/journal.pone.0231628)
Supplement: Attachment — Submitted filename: Response to Reviewers.docx [file pone.0231628.s003.docx]

Cáceres, February, 17^th^ 2020

Dear Daniel Boullosa,

The authors appreciate the time you spent to reading our manuscript and helping us to craft an improved version of the investigation. We are pleased to clarify your concerns, which we believe have improved the quality and applicability of this work. Please, find below our responses to each of your observations. We have made a concerted attempt to systematically address the specific concerns raised for this revision and we have highlighted the alterations to this revision within the manuscript in red for your convenience.

**REVIEWER:** “Thank you for your comment. We were looking at differences between male and female athletes since women’s participation is significantly lower at this race, and the number of research is lower for women [3]. Mujika Alberdi, A., García Arrizabalaga, I., and Gibaja Martíns, J. J. (2018). Impact of the behobia-san sebastián race on promoting healthy lifestyles. [Incidencia de la carrera Behobia-San Sebastián en el fomento de estilo de vida saludable] Apunts.Educacion Fisica Y Deportes, (131), 34-48. doi:10.5672/apunts.2014-0983.es.(2018/1).131.03.” Please also mention this paper and explanation in the manuscript.

**AUTHORS:** Thank you so much for your comment. We mentioned this paper (Lines: 47, 103) and we added the explanation in Lines: 102-104 “,and taking into account that women’s participation is significantly lower at the Behobia-San Sebastian race, and the number of research is lower for women [3] differences between male and female athletes will be considered”.

**REVIEWER:** “For instance, we find that intrinsic motivation
is associated with a higher incidence of injury. Therefore, it would be important that very
intrinsically motivated amateur athletes, having a good training plan, and being leaded by
professionals, so that, the association between their intrinsic motivation and incidence of
injury will be weaker, even when they push themselves hard”
Thank you for the explanation. Could you please change this in the abstract as well?

**AUTHORS:** We appreciate very mucho this indication and therefore, we have changed the information in the abstract section, and we have added the following information: “, i.e., it was found that intrinsic motivation was associated with a higher incidence of injury,” Lines 37,38, and in Lines 42-45: “Moreover, it is considered important that very intrinsically motivated amateur athletes, having a good training plan, and being leaded by professionals, therefore, the association between their intrinsic motivation and incidence of injury would be weaker, even when they push themselves hard”, completing thus the abstract.

**REVIEWER:** The cross-sectional manuscript. The relationship of recreational runners’ motivation and resilience levels to the incidence of injury: A mediation model concerns the relationship between resilience, motivation and injury rates. It is a current topic of interest in sports science and their writing, sample size and study-desing are generally sound. However, I recommend it to be rejected for publication for the following reasons:
The correlations observed in the study are not scientifically explained. Scientific evidence is lacking to support the assumption that matters in the present article (the enlightenment of how these physiological variables relate to injuries). Correlation does not mean causality. In my humble opinion, the discussion section does not meet the requirements and high standards of quality for a prestigious journal such as PlosOne.

**AUTHORS:** Thank you for your comment. Since the research line is very new, we are afraid that there is not easy to support scientific evidence about the obtained results in the literature, however, we added a paragraph in the limitation section. Lines: 291-293 “Likewise, another limitation was that scientific evidence was lacking due to the novelty of this topic, and physiological variables related to injuries were not considered." Previous research had associated psychological variables such as resilience or anxiety, or competitive level with athletes’ injuries [16,41], and these investigations have been considered during this manuscript.

16.Zurita-Ortega F, Chacón-Cuberos R, Cofre-Bolados C, Knox E, Muros JJ. Relationship of resilience, anxiety and injuries in footballers: Structural equations analysis. Ewen HH, editor. PLoS One. 2018;13: e0207860. doi:10.1371/journal.pone.0207860

44.Castro Sánchez M, Chacón Cuberos R, Zurita-Ortega F, Espejo Garcés T. Levels of resilience based on sport discipline, competitive level and sport injuries. RETOS-Nuevas Tendencias En Educ Fis Deport Y Recreacion. 2016; 162–165.

On other hand, we agree that correlation does not mean causality, in fact, we have corrected the manuscript in the previous review, changing causalities for associations, “We have revised the language of the manuscript and we have removed causalities, using instead associations, in line with the cross-sectional design of the study.” e.g. Lines:154-155, 313, 318-319, 365,366. Nevertheless, and following your concern, the cross-sectional design was considered a limitation of the study. Lines 291-293 “The cross-sectional study design was another limitation, as it did not allow causal relationships between the study variables to be established”.

Regarding the discussion section, we do not understand what we are supposed to improve, since we used the latest publications in prestigious journals like PLOS One, and we followed the way the discussion is organized in them. E.g: 12.	van der Worp MP, ten Haaf DSM, van Cingel R, de Wijer A, Nijhuis-van der Sanden MWG, Staal JB. Injuries in Runners; A Systematic Review on Risk Factors and Sex Differences. Zadpoor AA, editor. PLoS One. 2015;10: e0114937. doi:10.1371/journal.pone.0114937, 16.Zurita-Ortega F, Chacón-Cuberos R, Cofre-Bolados C, Knox E, Muros JJ. Relationship of resilience, anxiety and injuries in footballers: Structural equations analysis. Ewen HH, editor. PLoS One. 2018;13: e0207860. doi:10.1371/journal.pone.0207860, or 43. Codonhato R, Rubio V, Oliveira PMP, Resende CF, Rosa BAM, Pujals C, et al. Resilience, stress and injuries in the context of the Brazilian elite rhythmic gymnastics. Bergamini E, editor. PLoS One. 2018;13: e0210174. doi:10.1371/journal.pone.0210174

With respect to correlations comments’, the author would like to thank reviewers´ contributions, and we have decided to modify the redaction of correlations by: “In model 1, the number of injuries was not significant associated with resilience levels after being controlled for sex and age (β = -0.063; p = 0.089). However, in model 2 the number of injuries was positively associated with intrinsic motivation after being controlled for sex, age and resilience (β = 0.123; p < 0.05). In the other hand, there were not significant associations between the number of injuries and extrinsic motivation after the additional adjustment for intrinsic motivation in model 3 (β = -0.102; p = 0.072)”.

Thus, we hope that the information added is consistent with a scientific explanation. Please, if further information is needed, let us know, and we will attempt to include more scientific information.

Finally, and after having made all the modifications and comments discussed above, the authors appreciate the contributions developed by the reviewers, as they have helped us to improve the quality of the article. We hope that the comments regarding the contributions of the reviewers have been conducted correctly and the work may be of interest for later publication.

Please, if further changes are needed, just let us know and we would try to correct them as soon as possible.

We thank you in advance for your consideration,

Sincerely

Dr. Pedro Antonio Sánchez Miguel
